# The T7-Primer Is a Source of Experimental Bias and Introduces Variability between Microarray Platforms

**DOI:** 10.1371/journal.pone.0001980

**Published:** 2008-04-23

**Authors:** Ron M. Kerkhoven, Daoud Sie, Marja Nieuwland, Mike Heimerikx, Jorma De Ronde, Wim Brugman, Arno Velds

**Affiliations:** Central Microarray Facility, The Netherlands Cancer Institute, Amsterdam, The Netherlands; Max Planck Institute for Evolutionary Anthropology, Germany

## Abstract

Eberwine(-like) amplification of mRNA adds distinct 6–10 bp nucleotide stretches to the 5′ end of amplified RNA transcripts. Analysis of over six thousand microarrays reveals that probes containing motifs complementary to these stretches are associated with aberrantly high signals up to a hundred fold the signal observed in unaffected probes. This is not observed when total RNA is used as target source. Different T7 primer sequences are used in different laboratories and platforms and consequently different T7 primer bias is observed in different datasets. This will hamper efforts to compare data sets across platforms.

## Introduction

Amplification of messenger RNA is widely accepted as the method of choice to acquire sufficient copies of mRNA transcripts to enable microarray expression analysis of experimental samples [1], [2]. The method is robust, very reproducible and it yields high quality antisense RNA material which can be labeled with fluorescent dyes and hybridized to sense oligonucleotide microarrays. The differences in microarray data introduced by amplification are minimal [3], and are outweighed by the benefits of the amplification technique. When RNA sources are sufficiently available some laboratories prefer to hybridize un-amplified labeled RNA to microarrays [4]. Besides the mentioned discrepancies between total RNA and amplified RNA, additional variation in results is observed when the same amplified RNA sample is analyzed on different microarray platforms [5], [6]. Although the results are largely overlapping they are not completely identical and the reasons for this remain unclear. We have focused on resolving experimental bias associated with the T7 amplification protocol and the T7-primer sequence in particular. We assumed that the precise sequence of the T7 primer is important in this respect because the complete T7-primer is incorporated in the first strand cDNA generated by an RT enzyme upon annealing to the poly-A tail of mRNA extracted from samples.

**Figure 1:**
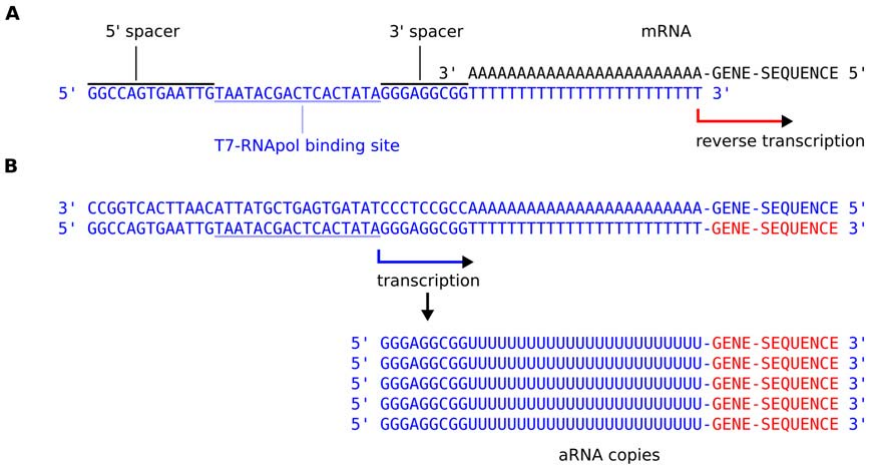
Schematic representation of the amplification of mRNA. A. Annealing of the oligo d(T) stretch of the Affymetrix T7 primer (blue) to the poly A tail of a mRNA molecule (black). Indicated are the location of the core T7 RNA polymerase binding site, the 5′ and 3′ spacer sequences flanking it and the start of reverse transcription (red) by the RT enzyme that produces the first stand cDNA incorporating the complete T7 primer sequence. B. Transcription start (blue arrow) directly 3′ from the RNA polymerase binding domain generates antisense RNA copies that have the 3′ spacer sequence of the T7 primer as a leader sequence at their 5′ side. Between this leader sequence and the gene sequences the copies have a stretch of uridine nucleotides that originate from the poly A tail of the mRNA.

Many versions of original “Eberwine” T7-primer sequence have been reported over the last decade [7], [8]. Some of these variations concern the length of the T stretch (ranging from 11–24 bases) or the use of one or more “anchor” nucleotides to direct the primer to the start of the poly-A tail of the mRNA as was applied by Agilent. A common feature of all of the T7 primers is the core T7-binding domain which is essential for the first interaction with the RNA polymerase and the start of transcription. The bases flanking the core T7 site are spacer sequences (fig 1A) that have been modified in attempts to improve the binding of the enzyme and the efficiency of the enzymatic reactions [9]. These spacer sequences have been introduced into the cDNA for different reasons. The 5′ spacer is introduced to avoid the T7 site to be distally located on the primer whereas the 3′ spacer in the original Eberwine primer is a reflection of the viral T7 sequence at that position which is thought to be important in the polymerase binding [7]. The general opinion today is that this spacer constitutes no more than a separator between the T7 site and the oligo-d(T) stretch.

We set out to investigate the consequence of the incorporation of the 3′ spacer sequence since in the final step in the amplification procedure it is transcribed by T7 RNA polymerase. As a consequence it becomes a leader sequence in all copies of amplified RNA (fig 1B). The 5′ spacer sequence is not present in amplification reaction products. Interestingly, different platforms have adopted different 3′ spacer sequences in their T7 primers. It is our hypothesis that these T7 spacer sequences may indeed cause hybridization artifacts on those probes containing complementary sequences to the spacer sequence (T7 3′ spacer motifs). We moreover suggest that variations in T7 spacer sequences introduce platform specific bias that may hamper the data comparison between platforms.

## Materials and Methods

### Statistical software

Statistics tests were run using the R-project software package [10].

### Datasets

To study the effect of the presence of motifs in a probe sequence we analyzed three data sources all using amplified RNA (I,II and III) and one data source using total RNA (IV):

- **“Operon (a)”** The compendium of experiments present in the database of the Central Microarray Facility of the Netherlands Cancer Institute (data table can be found in Table S1 and here: http://microarrays.nki.nl/research/T7/) comprising a total of 6657 human full genome Operon (version 3.0) oligonucleotide microarrays (http://microarrays.nki.nl/services/oligo.html).
- **“Agilent (a)”** Data obtained from a set of 295 human tumors analysed on 25 K oligonucleotide microarrays from Agilent/Rosetta [11].
- **“Affymetrix”, “Agilent (b)”, “Illumina” and “Operon (b)”** Datasets studied by the Microarray Quality Control project [6] containing microarray data sets obtained with amplified human RNA on the Affymetrix U133 Plus 2.0 arrays, the Agilent whole genome oligo microarrays (G4112A), Illumina Human-6 whole genome microarrays (Illumina; catalog # BD-25-101) and Operon V3.0 oligonucleotide platforms.
- **“Operon (c)” and “Operon (d)”** Studies on the Operon V3.0 oligonucleotide platform (from the NCI and NMC respectively) that were done without RNA amplification as described in the Microarray Quality Control project [6].

### T7 primers

**Figure 2:**
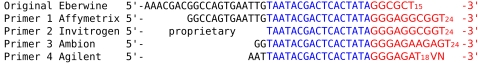
Primers studied. The core T7 polymerase binding site (in blue) and the 3′ spacer sequence (in red) are highlighted. Note that the 3′ spacer sequence in the T7 primers is not identical. In all cases the 3′ spacer is different from the Eberwine T7 primer sequence listed on top.

We set out to collect T7 primer sequences used in the field for which datasets are publicly available for analyses. We limited the study to the following T7-primers and looked in detail to their 3′spacer sequences relative to the original Eberwine primer sequence (fig 2) : The Affymetrix primer **(primer 1)**, also described as the “LinAmp1 primer” [7]. The Invitrogen primer **(primer 2)** - with partly disclosed sequence (W. Price, R.J. Rooney, personal communications) - is very similar (possibly identical) to the Affymetrix T7 primer. With respect to the 3′ spacer sequence primers **(1)** and **(2)** are identical. The Ambion primer **(primer 3)** having a 10 bp 3′spacer sequence that is different from the first two primers (B. Setterquist, personal communication). And finally the Agilent primer **(primer 4)** having a 6 bp long 3′ spacer sequence that is fully overlapping with the Ambion primer.

The 3′ spacer sequence from primers 1 and 2 is transcribed as a 9-mer leader sequence 5′GGGAGGCGG 3′, whereas the 3′ spacer of primer 3 is transcribed as a 10-mer sequence 5′GGGAGAAGAG 3′. Primer 4 introduces the short 6-mer leader sequence 5′GGGAGA 3′ into all copies of amplified RNA (fig 1). The motifs complementary to these leader sequences are 5′ CCGCCTCCC 3′, 5′ CTCTTCTCCC 3′ and 5′ TCTCCC 3′ respectively. Sequences that appear to be present in several probe designs of microarray platforms (table 1) indicate that no design rules exist today to preclude them. As a consequence these probes can hybridize to the leader sequence of every amplified RNA molecule in the hybridization mixture. A possible cause for cross reaction and biased measurements.

**Table 1:** Occurrence of sequence motifs in gene expression designs of different microarray platforms.

Primer	Motif	Operon (a)	Affymetrix
**1&2**	**CCGCCTCCC**	**14**	**4**
**1&2**	**CGCCTCCC**	**26**	**21**
**1&2**	**GCCTCCC**	**279**	**608**
**1&2**	**CCTCCC**	**1145**	**2831**

Primer	Motif	Operon (b)	Agilent (a)	Agilent (b)	Illumina
**3**	** CTCTTCTCCC **	**14**	**2**	**6**	**7**
**3**	**TCTTCTCCC**	**28**	**7**	**18**	**23**
**3**	**CTTCTCCC**	**101**	**31**	**73**	**110**
**3**	**TTCTCCC**	**295**	**104**	**246**	**291**
**3&4**	**TCTCCC**	**929**	**319**	**685**	**1050**

Table 1 presents an overview of the numbers of probes on different microarray platforms that share sequence overlap with the primer motif sequences that are used in combination with these platforms. Since primers sequences are transcribed into leader sequences of variable length we determined the abundance of complementary 6–10 bp long sequences in probes of the microarray platforms studied. Data in the table is given for the full length motifs and for the 3′ fragments thereof. Data on the abundance of all possible (internal) parts of the motifs and the percentages with respect to the total amounts of probes per platform can be found in the Table S2 and here: http://microarray.nki.nl/research/T7/.

Compared to the other platforms, the “Agilent (a)” platform has the lowest number of motif containing probes in their probe set: 1.3% (319 sequences) contain the relatively short 6-mer primer 4 motif TCTCCC. For “Agilent (b)” this is fraction has increased slightly to 1.6%. For “Operon” we find that 8.5% (2966 sequences) of the probes contain either the full 9-mer primer 1 motif or a 6-mer fragment of it. For the “Affymetrix” platform we find that 2,5% (3076 sequences) of all probe sequences and 37% of the 8204 probe sets contain a primer 1 motif sequence. On the Illumina platform 2% of the probe sequences contain the terminal 6-mer sequence TCTCCC from the primer 3 motif .

### Algorithms

#### Mean intensity of probes

Raw data (absolute intensities) was collected from 35K Operon arrays, printed in-house, and from 25K Agilent arrays and 22K Affymetrix U133 arrays from published literature. Using normalized hybridization data we calculated the mean probe intensity for each probe 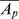 according to:
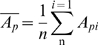
(1)Where n is the number of arrays and *A_pi_* the intensity of probe p in measurement i.

#### Mean intensity of motifs

The mean intensity per motif 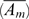 is calculated by averaging the mean probe intensities 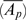 of the probes that contain the motif in question:
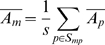
(2)Where S_p_ is the set of probes, S_mp_ the subset of S_p_ that contain motif m, s the size of S_mp_, n the number of measurements and 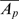 the mean intensity for probe p.

#### Intensity related motif bias

The intensity related motif bias is defined as the mean motif intensity divided by the standard deviation of the motif intensity:
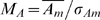
(3)

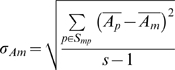
(4)


#### Rank related motif bias

Rank related motif bias is defined as the rank related motif intensity divided by the standard deviation of the rank related motif intensity:
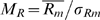
(5)

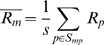
(6)

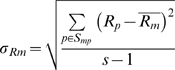
(7)Sampling the ranks limits the effect of outliers and enhances small differences that are observed with intensities.

## Results

### The T7 primer 3′ spacer motif causes an increase in spot intensity in a subset of spots

**Figure 3:**
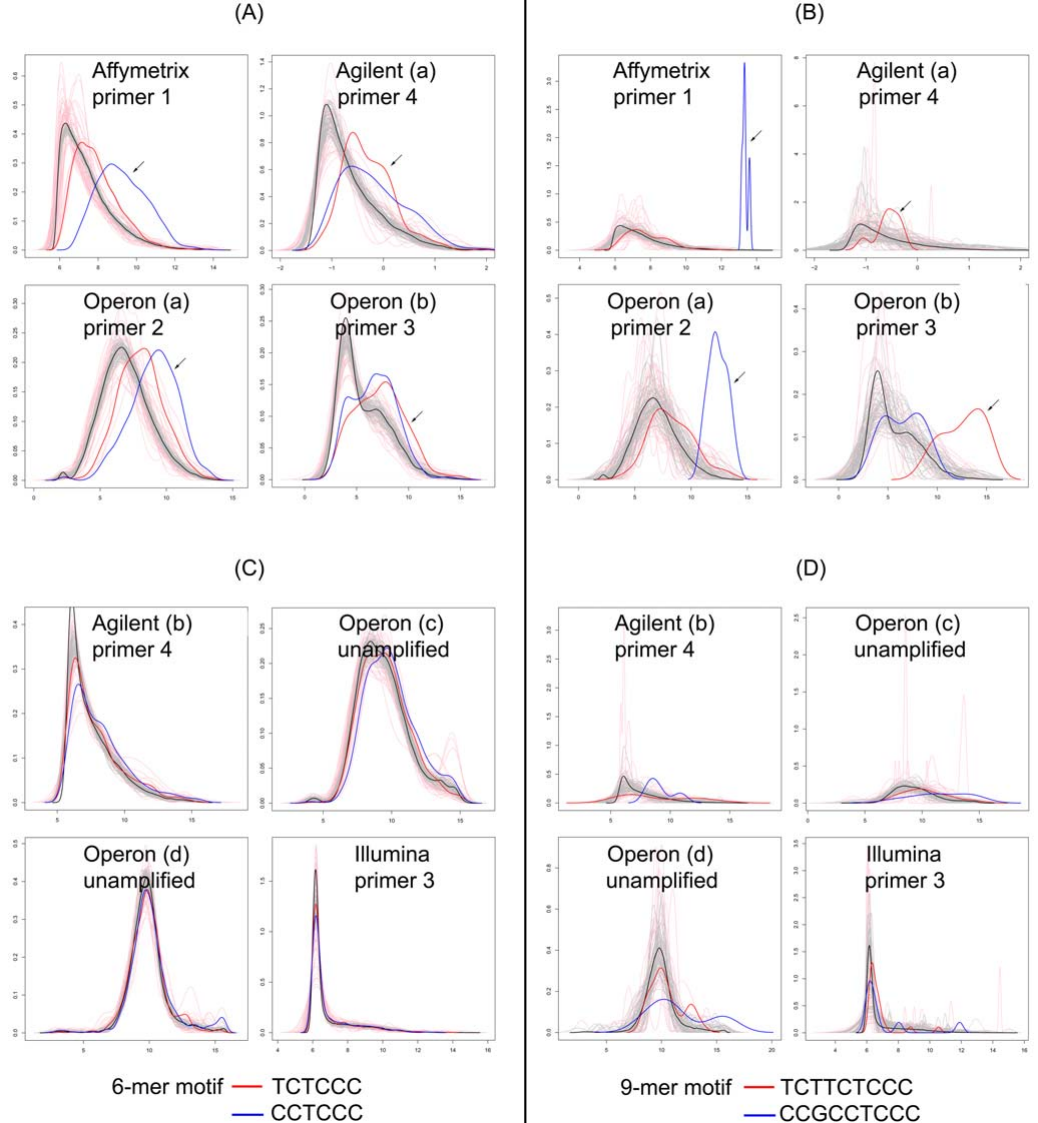
Density plots of oligonucleotide probes sets hybridized with RNA amplified with indicated T7 primers (listed in fig 2). Horizontal axis is the log intensity scale. A and B, platforms that show T7 primer 3′spacer sequence bias, C and D, platforms that show little or no T7 primer 3′spacer sequence bias. Density analyses were performed on oligonucleotides containing 6 mer sequences (A and C), and on 9-mer sequences (B and D) based on the 3′spacer sequences from primer 1 and 2 (blue) or primer 3 and 4 (red). Arrows indicate the right-shifted density lines for the probes having the 3′spacer sequence present in the primer that was used in the study (indicated at the top of the graphs). Control lines are plotted in black, grey or pink lines. The black line represents density data for all probes lacking T7 motifs; the pink lines are individual random motifs for 6-mer or 9-mer sequences (n = 50), and the grey lines represents data from individual random probes (n = equal to the size of the subset of probes containing the T7 motif used in the study).

To estimate the influence of the presence of T7 motif sequences in probes on the amount of amplified RNA that is observed to bind to these probes we calculated average intensity levels for subsets of probes. First, we selected those probes that do contain (parts of) the T7 motifs, and secondly we took those probes that do not contain such motifs (the ‘no-motif’ probes). For control purposes we also used subsets of probes either based on random selection of probes or on random motifs in probes. Finally, we compared the results obtained for primer motifs that were actually used in the studies under investigation (indicated with an arrow in fig 3 A,B) with motifs present in the primers that were not used in the studies but that closely resemble the primers used (overlap >80%).

The probes present in a ‘platform – T7 primer’ dataset were subdivided into subsets based on the motifs they contain. The discerning motifs used were the T7- 3′ spacer motifs listed in fig 2. For each probe an average intensity was calculated over all microarrays within the study (**mean probe intensity** 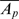, see Algorithms). Possible effects of 3′ spacer motifs are expected to be present in all hybridizations to a similar extent because all amplified RNAs of the set share the same leader sequence derived from the 3′ spacer of the T7 primer. For each subset of probes the average intensities were plotted as a density plot over the ^2^log intensity scale (fig 3). Where possible we performed these measurements for both the 6-mer and the 9-mer motifs. In all plots in figure 3, the control measurements for randomly selected probes (grey lines) or random sets of motifs (pink lines) consistently gave distributions that were close to the distribution of the no-motif probes (black lines).

For the probes of the “Operon (a)” dataset (fig. 3A, bottom left panel) with the 6-mer primer 2 motif CCTCCC a mean intensity of 9.13 is observed (blue line, arrow). This mean is clearly shifted up relative to the mean of the no-motif probes (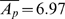, black line). This difference is even more pronounced if the same dataset is analyzed for the full 9-mer primer 2 motif (fig. 3B bottom left, blue line). A control subset of probes (fig 3A and B, bottom left panels, red line) selected from “Operon (a)” dataset containing the primer 4 motif TCTCCC (note that primer 4 was not used in these experiments) displays a smaller mean intensity shift about half the shift observed for the primer 2 motif probes. These curves clearly show a difference in mean intensity but since the underlying distributions are not normal, a different metric was needed to calculate a statistic for this (see ranked motif bias below).

Interestingly, shifts in mean probe intensities for data obtained from the same Operon V 3.0 probeset vary depending on the T7 primer used. For instance in the “Operon (b)” study (fig 3A and B bottom right panels), where primer 3 is used to amplify RNA we observe that primer 3 motifs shift the mean intensities furthest to the right, most pronounced in the 9-mer data (fig 3B bottom right panel). The shift observed for the 9-mer primer 2 motif probes in “Operon(a)” (fig 3B, bottom left, blue line) is almost absent in the “Operon(b)” data (fig 3B bottom right panel, blue line). Apparently the same Operon oligos behave differently in combination with a different primer indicating that the choice of the primer is causing these differences. If the comparison is carried out for datasets obtained again from the same Operon V3.0 platform but now using un-amplified RNA samples instead of amplified RNA, the differences between motif containing probes and their no-motif counterparts are minimal or absent (fig 3C,D upper right and bottom left panels), again stressing the idea that the T7 primer motif sequences are influencing intensities observed on affected probes.

On the somewhat older “Agilent (a)” platform we find similar but less pronounced shifts in distribution curves, which still boils down to a higher intensity for primer 4 motif containing probes. Note that the 9-mer motif CCGCCTCCC is absent in the “Agilent (a)” platform and therefore not plotted in figure 3B upper right panel. In the more recent G4112A Agilent arrays from the “Agilent (b)” study the shift is much less pronounced or absent (fig 3 C,D upper left panels).

The “Affymetrix” study uses primer 1, and again we observe a shift of 3.6 for the corresponding motif containing probes (fig 3A,B upper left panels, blue lines). If we study the primer 4 motif containing probes in the same dataset the shift is smaller (fig 3 A,B upper left panels, red lines). The Illumina platform uses primer 3 based amplification kits. From the distribution in fig 3 C and D (bottom right panels) we can see that there is no intensity shift in intensity for probes having a primer 3 motif. Also the other probe sets selected from the Illumina dataset show no differences in intensity profiles.

**Figure 4:**
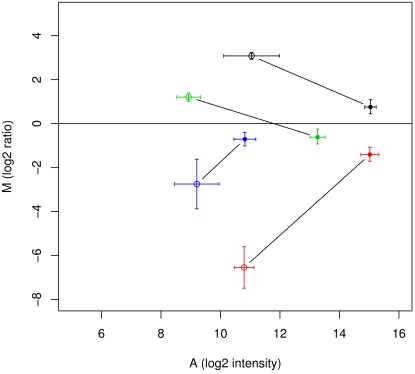
Significant ratios are lost when reporters are affected by T7 bias phenomenon. For this plot we used data from hybridizations that belong either to amplified RNA (closed circles) or unamplified RNA (open circles) in two different MAQC studies, “Operon (b)” and “Operon (d)” respectively. The “Operon (b)” study used primer 3 to amplify the total RNA. Plotted is the M versus A value for four probes that were selected from 61 probe sequences (all containing the 8-mer motif CTTCTCCC present in the primer used) because these four report a significant up or down regulation in the Operon(d) study. Indicated are the normalized and averaged MA values for microarrays (n = 10) hybridized with the same sample on the two platforms (error bars for standard deviation). The ratios observed for amplified RNA are situated closer to the zero level. From the individual microarray measurements 34 out of the 40 spots were a significant outlier in the unamplified RNA study. 22 (or 65%) of them lost the status of being a significant outlier after the amplification procedure. Operon Ids for the probes listed: H300004675 (black), H300006007 (red), H300008441 (green) and H300014662 (blue).

To investigate if the shift in mean intensities observed in amplified RNA studies affects the ratios observed for probes with T7 spacer sequences we compared two MAQC platforms, the “Operon (b)” and “Operon (d)” platform. Both types of microarrays use the same Operon oligonucleotide set and both were hybridized with identical samples. The only difference consists of using unamplified or amplified RNA. We selected probes from both platforms that were affected by T7 bias and compared the ratio measurements obtained for these probes. We observed that ratios for these probes are markedly closer to zero in the amplified RNA study compared to the unamplified RNA study (fig 4). As a result of this shift 22 out of the 34 (65%) gene expression measurements are no longer considered significant outliers under the Rosetta error model. Interestingly, this shift in ratios contrasts the general trend showing more extreme ratios for the probes not affected by the T7 bias in amplified RNA data. This is illustrated in Figure S3 that compares the spread of ratios between the unamplified RNA study (A and B) versus the amplified RNA study (C and D) in a box plot figure. When we focus on the unamplified RNA we observe that unaffected (A) and T7 bias affected probes (B) have very similar distributions. If we compare this to the amplified RNA distributions in C and D, we see that affected probes (D) show compressed distribution centering around 0. By comparing (A) and (C) one can furthermore conclude that amplification of RNA leads to increased ratios.

To better compare subsets of probes in a normalized manner and to allow for statistical evaluation we converted the intensity axis into a rank-axis. We ranked the mean probe intensities 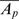 and assigned a rank number to each individual probe. We listed all possible 6-, 7-, 8- and 9-mer motifs present in the collection of probes of a platform. Each listed motif has an associated probe set, comprised of all probes containing that motif. We assigned to every motif the average rank of all probes in the corresponding probe set (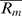). Motif CCTCCC (primer 1&2) appeared to be a significant outlier in the Affymetrix study (p = 1.4×10^−10^), the “Operon (a)” study (p = 9.4×10^−10^), the “Agilent (a)” study (p = 2.4×10^−7^) and the “Operon (b)” study (p = 4.7×10^−6^). Surprisingly, this motif also was an outlier in the “Operon(c)” study (p = 3.9×10^−3^). Motif TCTCCC however, (primer 3&4) was a significant outlier in the Affymetrix study (p = 5.2×10^−3^), the “Agilent (a)” study (p = 9.9×10^−7^), the “Operon (a)” study (p = 1.8×10^−4^ ) and the “Operon (b)” study (p = 3.9×10^−9^). The 9-mer motifs showed a very similar trend. An overview of all density plots and the associated p-values is shown in Table S3 and Figure S2. Except for the previously mentioned CCTCCC motif in the “Operon(c)” study, none of the motifs was a significant outlier in the Illumina study, the “Agilent (b)” study and the studies using unamplified RNA, “Operon (c)” and “Operon (d)”.

### Motifs that are associated with intensity bias can be identified in any probe within any platform by measuring the Rank related motif bias

**Figure 5:**
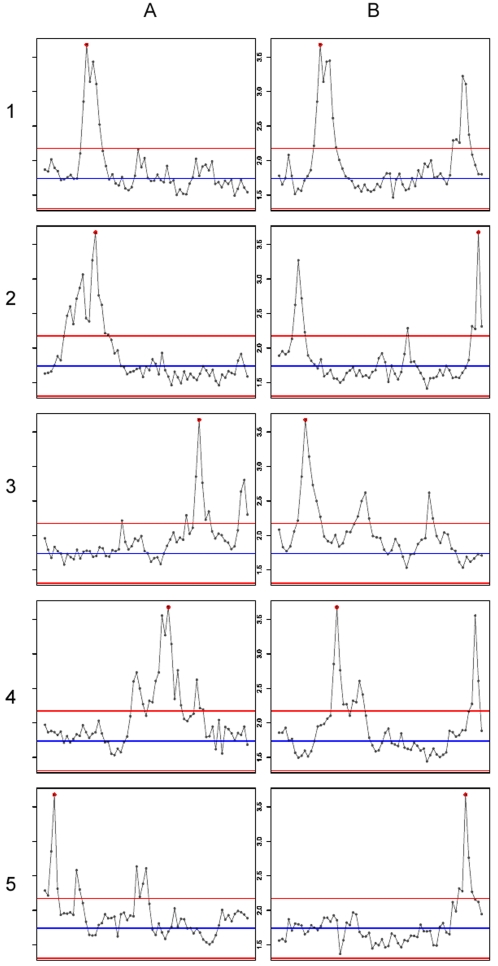
Examples of oligonucleotide probes from the “Operon (a)” probe set where T7 bias motifs are identified by plotting the rank related motif bias (Mr). Column A and B: Ten examples plots for probe sequences that contain the T7primer 1 3′ spacer motif CCTCCC. The 70-mer probe sequence is split in 6-mer fragments over the x-axis and the intensity rank related motif bias (Mr) is plotted on the y-axis. The red dot represents the position of the CCTCCC motif in the probe sequence. The blue line is the average over all the motifs from a probe sequence, the red lines represent the standard deviation up and down for the same measurement over all 6-mers in the whole probe. Operon Ids for the probes listed A1 to B5 and C1 to D5: H200018976, H300020020, H200016156, H300007790, H300013997, H300022725, H200010013, H300019787, H200001204, H200004220.

The T7 primer 3′ spacer sequence motifs are expected to have high mean intensities and therefore have a high mean probe rank. However, motifs that are exclusively part of abundantly expressed genes will also have high mean probe intensities and ranks. To discriminate motifs associated with highly abundant genes from those associated with bias motifs we reasoned that variations in observed 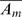 will be high for the first and low for the latter motifs. Motifs present in highly abundant genes are likely to also be present in low-abundant genes. It is unlikely that motifs in highly abundant genes are the reason for the high abundance of that mRNA. We expect therefore that the standard deviation over the observed motif intensity (σ*_Am_*) is high for abundant mRNAs and low for bias motifs (like the T7 motifs). If the motif really is the reason for the high intensity bias, the motif will invariably be associated with high 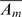 values and subsequently have a low σ*_Am_*. By calculating the **intensity related motif bias** (*M_A_*) by dividing the 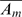 by the σ*_Am_*, and the **rank related motif bias** (*M_R_*) by dividing the 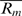 by σ*_Rm_* we introduced two methods for detecting motifs with associated intensity bias. The **rank related motif bias** (*M_R_*) showed to be superior in separating bias motifs from background motifs. An example of this is displayed in fig 5 where a random selection of 10 primer-motif containing probes is taken from the “Operon (a)” study and analyzed for the *M_R_* value. All primer related 3′ spacer sequence motifs could be traced back to peaks of rank related motif bias. Some additional peaks were also observed. These represent biases other than T7 primer related biases. To rule out that the peaks found in fig 5 are the result of motifs present in highly abundant genes, we plotted our *M_R_* data against SAGE data of highly abundant genes. Out of the full repository of SAGE data from NCBI (ftp://ftp.ncbi.nlm.nih.gov/pub/sage/extr/tag_lib_freq.zip) and (http://www.ncbi.nlm.nih.gov/SAGE) the top 100 highest abundant gene products present in 4090 SAGE datasets were selected. For each motif we determined the fraction of abundant SAGE genes that are targeted by the corresponding subset of probes (F_SAGE_). There is no significant correlation between F_SAGE_ versus the *M_R_* of the motifs (see Figure S1 and http://microarray.nki.nl/research/T7/). We can thus conclude that the *M_R_* calculation is a good method for finding motifs linked to aberrant high signals. Moreover, the method is not hampered by motifs belonging to highly expressed genes.

## Discussion

The T7 primer used in amplification of mRNA for microarray applications is a potential source for bias in microarray data. The bias appears to be common to most microarray platforms that use amplification and mean intensities of one to nine percent of the probes increase aberrantly to levels up to a hundred fold the average level of unaffected probes. It is also dependent on the sequence of the T7 primer and it is absent from hybridizations where unamplified (total) RNA is used.

We find that the older Agilent, the Operon V3.0 and the Affymetrix data sets are most affected and that the newer Agilent and Illumina platforms are virtually free from the phenomenon.

The T7 bias can be pinned down to a precise sequence domain of the T7 primer used in the amplification, the 3′ spacer sequence. This sequence is introduced by the amplification procedure as a leader sequence at the 3′ terminus of *all* amplified (and subsequently labeled) molecules. As a consequence, in microarray hybridizations using this amplified RNA, these motifs are abundantly present. Apparently this high concentration drives probes with complementary sequences to bind amplified RNA, irrespective of the gene sequence present in the amplified RNA. This leads to a change in ratio for these probes more reflecting the total amounts of red and green in the hybridization mixture which, under normal circumstances, are present in equal amounts (1∶1). True gene expression differences therefore become masked by the T7 bias (see fig 4 and Figure S3).

The bias has gone unnoticed until now, most likely because it is restricted to a subset of the features on the microarray, namely only those that contain motifs complementary to the 3′ spacer sequence of the T7 primer. Melting temperatures of the short motif sequences fall below the hybridization temperature so in theory these motifs are unable to hybridize. In addition, previous findings indicated that short (linker) sequences do not introduce hybridization artifacts on microarrays [12]. The apparent contradiction with our results might be explained by the differences in the complexities of the linker pools. Complex linker mixtures may react differently to a single 6–9 mer T7 primer motif sequence. This may also have contributed to their concealment.

The intensity measured from a feature consists of two components: the gene expression and the bias. To which extent the T7 bias influences the measured ratio, assuming that the bias signal is equal for every affected feature, therefore depends on the signal coming from gene expression. As mentioned, high bias signals shift the log-ratio measured in affected features on dual color platforms towards 0 (no difference measured) and the spot is therefore easily lost from the analysis because it will not be selected as outlier anymore. In single channel measurements, on the contrary, T7 biased motifs features become over-estimations of gene expression because of the fact that the aberrant motif bias signals add up to the true gene expression signals. Here, the spots might have been noticed as consistently up regulated. Still, as in the case of Affymetrix, the spot is likely to become an outlier in the probe-set used to detect a single transcript and will therefore be discarded from the estimation of the averaged probe intensity for the transcript. Looking at actual probe data versus gene data confirms this assumption (data not shown).

Our results clearly show that motifs present in the 3′ spacer sequences of T7 primers correlate strongly with the intensity bias observed on probes with complementary sequences and can induce intensity shifts up to a hundred fold the average level of unaffected probes. It is remarkable that the shift in mean probe intensity for the selected probes in fig 3 is so specific to the platform-primer combination studied. Relatively small variations in the 3′ spacer sequences of the T7 primers immediately reduce the phenomenon dramatically. This can for instance be seen in fig 3A where a much smaller shift in mean probe intensity is observed for those populations of probes that carry motifs from related, nearly identical T7 primers. These curves are based on probes carrying motifs that vary only by a single base compared to the primer sequences that were actually used in the studies (marked by an arrow). A possible cause for the fact that we still measure a shift for a probe sequence not used in the study is the overlap among the motifs (CCTCCC and TCTCCC) taken from the 3′ spacer sequences of the primers (see fig 2).

The absence of an apparent intensity shift for subsets of probes containing complementary primer motifs in the “Agilent (b)” and “Illumina” platforms (see fig 3) is remarkable since these platforms do use amplified RNA and primers with motifs that might induce T7 bias in the data. Apparently these platforms have found ways to reduce the bias effects. For example, additives in the hybridization mixtures can be used to prevent the T7 bias (see below).

Several oligonucleotide designs for gene expression analysis have been generated with probes containing motifs complementary to 3′ spacer sequences of the T7 primers. Improvements to prevent the occurrence of T7 motif bias can be found along two lines. One option is to eliminate sequence overlap from the T7 primers. In that case the 3′ spacer sequence could be completely removed or replaced by an uncomplimentary sequence. It is unclear at the moment if this is feasible in the sense that we don't know if a T7 enzyme will be functional using such a drastically modified primer. On the other hand, probes can be designed that lack complementary sequences to these T7 spacer motifs and thus eliminate the artifact. In the mean time, one could make use of specific blocking oligos that react with the motif sequences on the targets or on the probe sequences. Preliminary results (M.N., R.M.K. and M.H) where single stranded oligos with complementary sequences to T7 spacer domains or to T7 motif domains from probe sequences were added to hybridization mixtures indicate that blocking oligos indeed reduce the T7 bias phenomenon. In fact, the approach is similar to the one taken to minimize side-effects of the stretch of T-nucleotides in the amplified RNA (a remnant of the poly(A) tail of the mRNA) by adding poly-d(A) in the hybridization buffer.

The aberrant high signals we observe on features displaying T7-bias motifs are the result of excess binding of labeled target molecules. Our data show that this is the result of binding of the 3′spacer of these molecules to probes on the array. We hypothesize that the binding we observe is a reflection of the molar excess of the amplified material available for binding. Since *all* target molecules carry the 3′ spacer sequence, the hybridization kinetics on the features containing a complementary T7-bias domain (1 to 5% of the probe sequences) may be forced into the binding state. A secondary feature of the T7-bias domains is that they are GC-rich and distally located on the molecule which may increase binding affinity.

The method we describe for the calculation of motif bias is capable of detecting probes that are affected by T7 based intensity artifacts. Clearly, the 3′ spacer sequence in the T7 primer is associated with a higher than average rank related motif bias. It is however not the only motif that has a high value in this respect (see fig 5). Until now, the T7 spacer motifs are the only motifs for which we discuss an explanation because we can directly link the observed bias sequence back to the T7 primer sequence. There are however also other motifs, unrelated to the T7 primer sequence, that score high in the *M_R_*-value calculation. We are currently investigating the nature and experimental causes for these other high *M_R_*-value associated motifs observed in the datasets under study.

## Supporting Information

Table S1 Condensed ratio table. Data table where we indicate the Operon Identifier (oligoID), the Central Microarray Identifier (reporterID), the mean of intensities observed for this probe over the data in the CMFdatabase (mean) and the statistical values associated.
(5.66 MB TXT)

Table S2 Table of motif containing probes per platform.
(0.03 MB DOC)

Table S3 Statistical values (p-values) reflecting the chance that a particular motif belongs to a distribution of ranked motifs.
(0.03 MB DOC)

Figure S1 M(R) score versus SAGE scores Figure showing that there is no correlation between the presence of a motif in a probe that binds abundant mRNAs and the T7-bias M(R) score of that motif. Dotplot of the SAGE tag-score versus the M_R_ of the motifs. SAGE data from NCBI (ftp://ftp.ncbi.nlm.nih.gov/pub/sage/extr/tag_lib_freq.zip) and (http://www.ncbi.nlm.nih.gov/SAGE). A selection made of the top 100 highest abundant (and therefore best characterized signals in SAGE) gene products present in 4090 SAGE datasets. Their sequence was split in motifs of different lengths and plotted against the M_R_ of the same motifs. Panels A to D: plots for motifs that are 6, 7, 8 and 9-mers in length. Notice that there is no correlation between the two aspects of the same motif.
(0.27 MB DOC)

Figure S2 Density plots of the motif mean ranked intensity for all 6-mer (A,C) and 9-mer (B,D) motifs in the platforms studied. Indicated is the position of the T7 bias motifs CCTCCC and TCTCCC that are significant outliers (p-values in supplemental data 3) in the studies that used the respective T7 primers (1&2 and 3&4, panel A). In the studies listed in panel C, these motifs aren't found as outliers (except for CCTCCC in “Operon (c)”. The corresponding 9-mer motifs show a very similar trend (B,D).
(2.63 MB TIF)

Figure S3 Boxplot representation of the ratio distribution observed from an unamplified RNA study “Operon (d)” (A and B) versus an amplified RNA study “Operon (b)” (C and D). The spread of the ratios is shown for the T7 bias unaffected probes (A and C) versus the affected probes (B and D). For unamplified RNA an equal distribution is present for unaffected (A) and T7 bias affected probes (B). For amplified RNA however, the T7 bias affected probes (D) display a clear reduction in ratio distribution compared to the unaffected probes (C). Comparing A and C indicate that amplification in itself leads to increased ratio levels.
(3.66 MB TIF)

## References

[1] Van Gelder RN, von Zastrow ME, Yool A, Dement WC, Barchas JD (1990). Amplified RNA synthesized from limited quantities of heterogeneous cDNA.. Proc Natl Acad Sci U S A.

[2] Petalidis L, Bhattacharyya S, Morris GA, Collins VP, Freeman TC (2003). Global amplification of mRNA by template-switching PCR: linearity and application to microarray analysis.. Nucleic Acids Res.

[3] Wang E, Miller LD, Ohnmacht GA, Liu ET, Marincola FM (2000). High-fidelity mRNA amplification for gene profiling.. Nat Biotechnol.

[4] Petersen D, Chandramouli GV, Geoghegan J, Hilburn J, Paarlberg J (2005). Three microarray platforms: an analysis of their concordance in profiling gene expression.. BMC Genomics.

[5] Kuo WP, Liu F, Trimarchi J, Punzo C, Lombardi M (2006). A sequence-oriented comparison of gene expression measurements across different hybridization-based technologies.. Nat Biotechnol.

[6] Shi L, Reid LH, Jones WD, Shippy R, MAQC Consortium (2006). The MicroArray Quality Control (MAQC) project shows inter- and intraplatform reproducibility of gene expression measurements.. Nat Biotechnol.

[7] Baugh LR, Hill AA, Brown EL, Hunter CP (2001). Quantitative analysis of mRNA amplification by in vitro transcription.. Nucleic Acids Res.

[8] Luo L, Salunga RC, Guo H, Bittner A, Joy KC (1999). Gene expression profiles of laser-captured adjacent neuronal subtypes.. Nat Med.

[9] Nacheva GA, Berzal-Herranz A (2003). Preventing nondesired RNA-primed RNA extension catalyzed by T7 RNA polymerase.. Eur J Biochem.

[10] R_Development_Core_Team R (2007). A Language and Environment for Statistical Computing, R.F.f.S..

[11] van de Vijver MJ, He YD, van't Veer LJ, Dai H, Hart AA (2002). A gene-expression signature as a predictor of survival in breast cancer.. N Engl J Med.

[12] He Z, Wu L, Li X, Fields MW, Zhou J (2005). Empirical establishment of oligonucleotide probe design criteria.. Appl Environ Microbiol.

