# Supplementary material for: The T7-Primer Is a Source of Experimental Bias and Introduces Variability between Microarray Platforms
Source: PLoS One. 2008 Apr 23;3(4):e1980. doi: 10.1371/journal.pone.0001980 (PMC2292241; doi:10.1371/journal.pone.0001980)
Supplement: Table S2 — Table of motif containing probes per platform. (0.03 MB DOC) [file pone.0001980.s002.doc]

**SUPPLEMENTAL DATA 2**

Table of motif containing probes per platform.
