# Supplementary material for: The T7-Primer Is a Source of Experimental Bias and Introduces Variability between Microarray Platforms
Source: PLoS One. 2008 Apr 23;3(4):e1980. doi: 10.1371/journal.pone.0001980 (PMC2292241; doi:10.1371/journal.pone.0001980)
Supplement: Table S3 — Statistical values (p-values) reflecting the chance that a particular motif belongs to a distribution of ranked motifs. (0.03 MB DOC) [file pone.0001980.s003.doc]

**SUPPLEMENTAL DATA 3**

Statistical values (p-values) reflecting the chance that a particular motif belongs to a distribution of ranked motifs.
