# Supplementary material for: The T7-Primer Is a Source of Experimental Bias and Introduces Variability between Microarray Platforms
Source: PLoS One. 2008 Apr 23;3(4):e1980. doi: 10.1371/journal.pone.0001980 (PMC2292241; doi:10.1371/journal.pone.0001980)
Supplement: Figure S1 — M(R) score versus SAGE scores Figure showing that there is no correlation between the presence of a motif in a probe that binds abundant mRNAs and the T7-bias M(R) score of that motif. Dotplot of the SAGE tag-score versus the MR of the motifs. SAGE data from NCBI (ftp://ftp.ncbi.nlm.nih.gov/pub/sage/extr/tag_lib_freq.zip) and (http://www.ncbi.nlm.nih.gov/SAGE). A selection made of the top 100 highest abundant (and therefore best characterized signals in SAGE) gene products present in 4090 SAGE datasets. Their sequence was split in motifs of different lengths and plotted against the MR of the same motifs. Panels A to D: plots for motifs that are 6, 7, 8 and 9-mers in length. Notice that there is no correlation between the two aspects of the same motif. (0.27 MB DOC) [file pone.0001980.s004.doc]

**SUPPLEMENTAL DATA 4**

Figure showing that there is no correlation between the presence of a motif in a probe that binds abundant mRNAs and the T7-bias *M(R)* score of that motif.


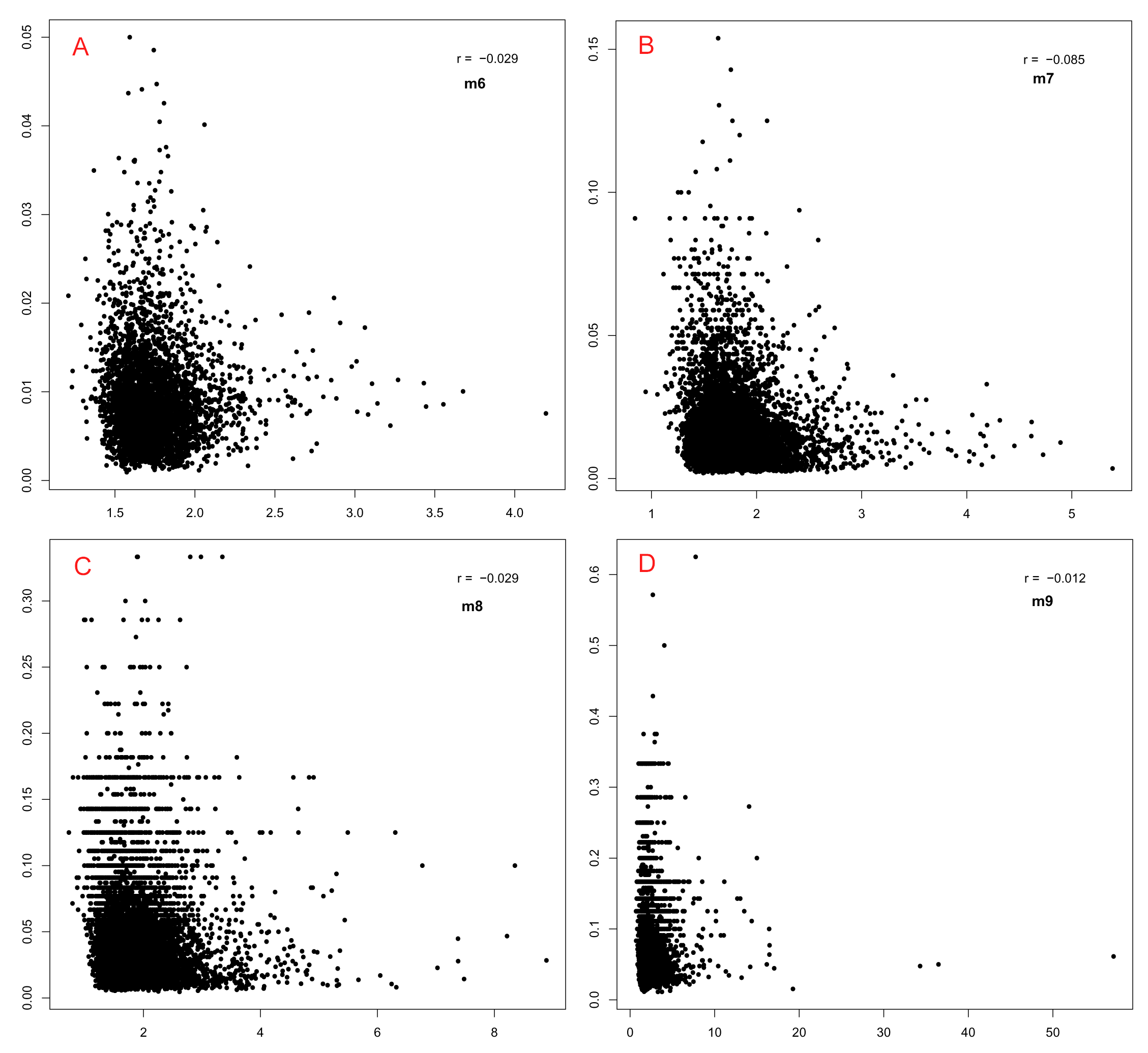


Dotplot of the SAGE tag-score versus the $M_{R}$ of the motifs. SAGE data from NCBI (<ftp://ftp.ncbi.nlm.nih.gov/pub/sage/extr/tag_lib_freq.zip>) and (http://www.ncbi.nlm. nih.gov/SAGE). A selection made of the top 100 highest abundant (and therefore best characterized signals in SAGE) gene products present in 4090 SAGE datasets. Their sequence was split in motifs of different lengths and plotted against the $M_{R}$ of the same motifs. Panels A to D: plots for motifs that are 6, 7, 8 and 9-mers in length. Notice that there is no correlation between the two aspects of the same motif.
