# Supplementary figures and images for: The T7-Primer Is a Source of Experimental Bias and Introduces Variability between Microarray Platforms
Source: PLoS One. 2008 Apr 23;3(4):e1980. doi: 10.1371/journal.pone.0001980 (PMC2292241; doi:10.1371/journal.pone.0001980)

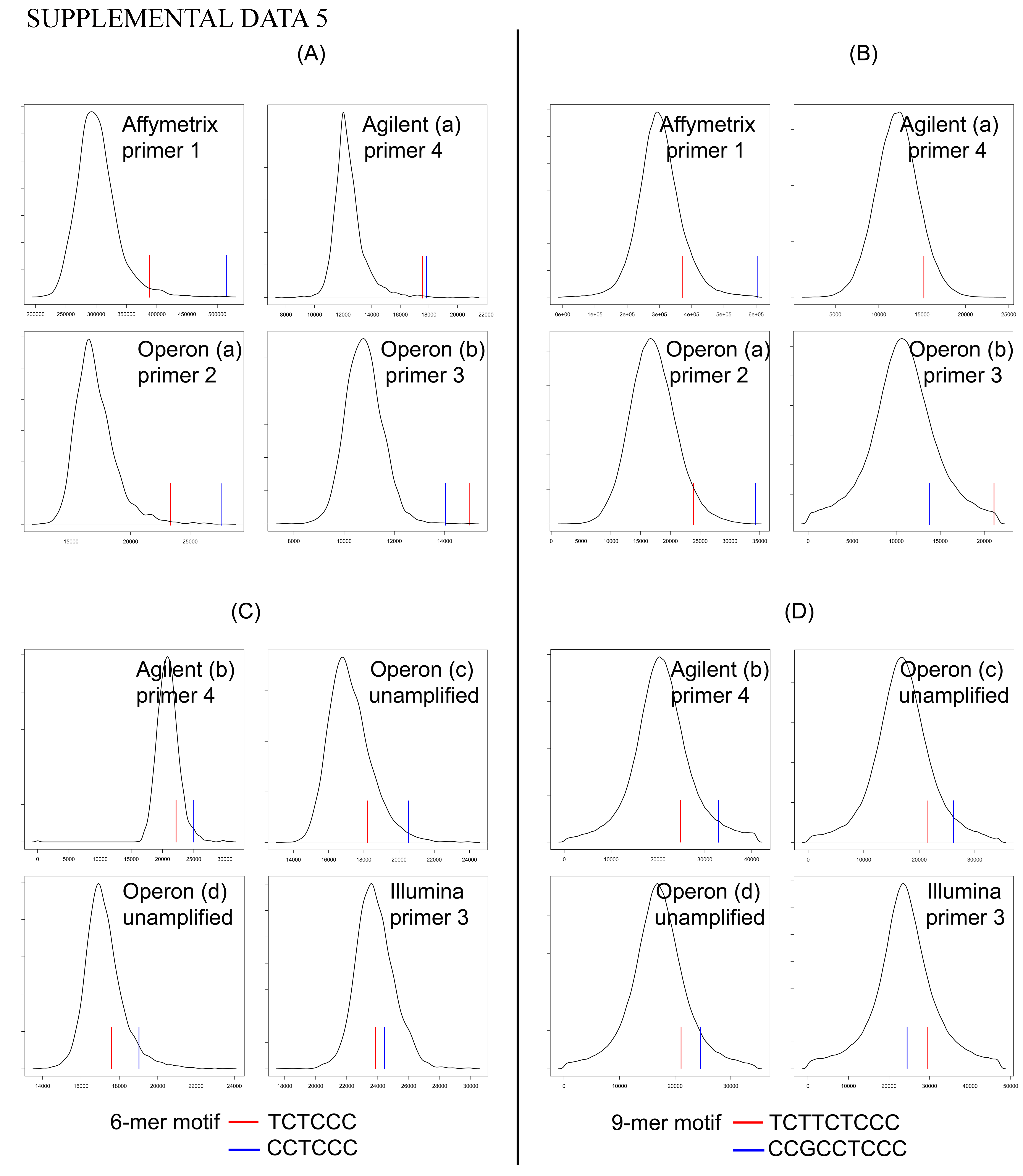

Supplement: Figure S2 — Density plots of the motif mean ranked intensity for all 6-mer (A,C) and 9-mer (B,D) motifs in the platforms studied. Indicated is the position of the T7 bias motifs CCTCCC and TCTCCC that are significant outliers (p-values in supplemental data 3) in the studies that used the respective T7 primers (1&2 and 3&4, panel A). In the studies listed in panel C, these motifs aren't found as outliers (except for CCTCCC in “Operon (c)”. The corresponding 9-mer motifs show a very similar trend (B,D). (2.63 MB TIF) [file pone.0001980.s005.tif]
